# Polymorphism’s MBOAT7 as Risk and MTARC1 as Protection for Liver Fibrosis in MASLD

**DOI:** 10.3390/ijms26136406

**Published:** 2025-07-03

**Authors:** Sofia Rocha, Claudia P. Oliveira, José Tadeu Stefano, Roberta P. Yokogawa, Michele Gomes-Gouvea, Patricia Momoyo Youshimura Zitelli, Joyce Matie Kinoshita Silva-Etto, Eduarda Donegá Martins, Mario G. Pessoa, Flavio F. Alcantara, Raymundo S. Azevedo, João Renato Rebello Pinho

**Affiliations:** 1 Laboratório de Gastroenterologia e Hepatologia Tropical—LIM07, Faculdade de Medicina da Universidade de São Paulo (FMUSP), São Paulo 05508-000, SP, Brazil; 00sofiarocha@gmail.com (S.R.); michele.gouvea@hc.fm.usp.br (M.G.-G.); joyce.mksilva@hc.fm.usp.br (J.M.K.S.-E.); eduarda.donega19@gmail.com (E.D.M.); 2 Laboratório de Gastroenterologia Clínica e Experimental—LIM07, Departamento de Gastroenterologia, Hospital das Clínicas Faculdade de Medicina da Universidade de São Paulo (HCFMUSP), São Paulo 05403-000, SP, Brazil; cpm@usp.br (C.P.O.); jose.tadeu@fm.usp.br (J.T.S.); patricia.momoyo@hc.fm.usp.br (P.M.Y.Z.); mgpessoa@usp.br (M.G.P.); 3 Departamento de Patologia, LIM/03-Laboratório de Medicina Laboratorial, Hospital das Clínicas, Faculdade de Medicina da Universidade de São Paulo, São Paulo 05403-000, SP, Brazil; roberta.yokogawa@hc.fm.usp.br (R.P.Y.); flavio.alcantara@hc.fm.usp.br (F.F.A.); 4 Departamento de Patologia-LIM01, Faculdade de Medicina da Universidade de São Paulo, São Paulo 05508-000, SP, Brazil; razevedo@usp.br; 5 Hospital Israelita Albert Einstein, São Paulo 05652-900, SP, Brazil

**Keywords:** liver fibrosis, MASLD, PNPLA3, MBOAT 7, MTARC1, molecular biology

## Abstract

Previous large-scale genetic studies identified single-nucleotide polymorphisms (SNPs) of the membrane bound O-acyltransferase domain containing 7 (MBOAT7) and patatin-like phospholipase domain containing 3 (PNPLA3) genes as risk factors for metabolic dysfunction-associated steatotic liver disease (MASLD). However, this has not yet been investigated in Brazilian patients. In this study, we evaluated the association between the PNPLA3 variant rs738409 and MBOAT7 variant rs641738 and the risk of hepatic fibrosis or liver cirrhosis in MASLD etiology. In parallel, we also aimed to evaluate a protective SNP of the mitochondrial amidoxime-reducing component 1 (MTARC1) gene. We also evaluated TM6SF2 rs58542926, GCKR rs1260326 and rs780094, and HSD17B13 rs72613567 and they were not associated with liver fibrosis. The study was conducted at the Department of Gastroenterology and Nutrology, Hospital das Clínicas da Faculdade de Medicina da Universidade de São Paulo (HCFMUSP), and included 113 patients with liver fibrosis (F0–F1), 99 patients with significant liver fibrosis (F2–F4), and 90 controls. SNPs were genotyped by quantitative PCR, using TaqMan allelic discrimination assays. Overall, the PNPLA3 GG genotype was more frequent in F2–F4 (23%) and F0–F1 (22%) patients than in controls (9%; *p* = 0.02). The MBOAT7 TT genotype was significantly associated with fibrosis, with a prevalence of 23% in F2–F4 patients versus 10% in F0–F1 and 11% in controls (*p* = 0.01). This association was confirmed by regression analysis (OR = 5.01 95% CI: 1.86–13.49; *p* = 1.41 × 10^−3^). The protective MTARC1 AA genotypes were more frequent in controls (52%) when compared to patients with fibrosis (5% *p* = 2.76 × 10^−20^).

## 1. Introduction

Metabolic dysfunction-associated steatotic liver disease (MASLD), formerly known as non-alcoholic fatty liver disease (NAFLD), has become the most prevalent cause of chronic liver disease worldwide, with an estimated global prevalence of approximately 30% [1,2,3]. This increase is closely associated with the rising incidence of obesity, insulin resistance, type 2 diabetes mellitus, and other features of metabolic syndrome [4,5,6]. The recent change in nomenclature to MASLD reflects a more accurate understanding of its pathogenesis, which is strongly linked to metabolic dysfunction, rather than simply the absence of alcohol consumption [1]. Despite this shift, MASLD encompasses a broad histological spectrum ranging from simple steatosis to steatohepatitis with significant and advanced fibrosis, which significantly increases the risk of cirrhosis, hepatocellular carcinoma (HCC), and liver-related mortality [7,8].

In recent years, genetic susceptibility has emerged as a crucial component in the development and progression of MASLD as polymorphisms (SNP—single nucleotide polymorphism) in the patatin-like phospholipase domain containing 3 (PNPLA3), transmembrane 6 superfamily member 2 (TM6SF2), glucokinase regulator (GCKR), membrane bound O-acyltransferase domain containing 7 (MBOAT7), hydroxysteroid 17-beta dehydrogenase 13 (HSD17B13), and mitochondrial amidoxime-reducing component 1 (MTARC1) genes, which regulate lipid metabolism and liver inflammation [9]. The rs738409 (Ile148Met) polymorphism in the PNPLA3 gene is one of the most studied and is associated with increased hepatic triglyceride accumulation, promoting a greater risk of inflammation and fibrosis. Similarly, variants in the MBOAT7 (rs641738) gene have been linked to worsening liver fibrosis and progression to HCC [10,11]. In contrast, the MTARC1 (rs2642438) gene has been described as a protective factor against inflammation and fibrogenesis, reducing the risk of MASLD progression to advanced stages of the disease [12,13]. The literature surrounding the validation of polymorphisms as risks and protective factors for liver fibrosis in MASLD has evolved significantly over the past few years.

Despite the well-established role of these genetic variants, most studies have focused on populations of European or Asian ancestry, with limited data available for admixed populations such as those in Brazil. Given the country’s high degree of genetic heterogeneity, investigating the frequency and clinical impact of these SNPs in Brazilian individuals is essential to improve risk stratification and promote personalized approaches to MASLD management. Thus, understanding the impact of these genetic factors on the progression of MASLD can contribute to the development of personalized risk stratification and clinical management strategies, optimizing the early identification of individuals with a greater predisposition to significant and advanced fibrosis.

Research from the Department of Gastroenterology and Nutrology, Hospital das Clínicas da Faculdade de Medicina da Universidade de São Paulo (HCFMUSP), which began in October 2023, has provided fundamental insights into the role of genetic factors in MASLD, identifying the MBOAT7 rs641738 (TT) and PNPLA3 rs738409 (GG) variants as risk factors for fibrosis and MTARC1 rs2642438 as a protective variant. The analyses highlighted the association of variants related to lipid metabolism with the accumulation of fat in the liver and histological severity, establishing a link between genetic predisposition and the progression of the disease.

The data reinforced the association of the PNPLA3-GG and MBOAT7-TT polymorphisms with serious liver injury and fibrosis, particularly in the admixed population. The findings were consistent with studies of the non-Hispanic Latin American population, thus broadening the understanding of the function of these genes in diverse demographic groups and contributing to the discourse on liver fibrosis. Adding to this narrative, protective genetic factors that influence liver disease were explored, including the MTARC1-GG gene, suggesting a multifactorial approach to understanding MASLD.

## 2. Results

We analyzed 212 patients with MASLD confirmed by liver biopsy, divided into two groups: Group 1 (F0/F1, n = 110) and Group 2 (F2/F3/F4, n = 102). Ninety individuals without a diagnosis of liver disease were included as controls. The mean age was similar between the groups (52.8 ± 10.91 vs. 55.7 ± 11.46 years), with a predominance of women (68.9%). BMI was significantly higher in Group 1 (32.29 ± 6.49 kg/m^2^ vs. 30.8 ± 5.98 kg/m^2^; *p* < 0.01). Group 3 (control) had a mean age of 37.8 ± 13.57 years, with males predominating in 53.33% of cases. BMI was 24.05 ± 3.49 kg/m^2^. No laboratorial assays were carried out in this control group consisting of candidates for blood donors.

Biochemically, Group 2 had significantly higher levels of AST (49.81 ± 40.24 vs. 31.95 ± 26.90 U/L; *p* < 0.001), ALT (58.16 ± 42.26 vs. 44.34 ± 35.72 U/L; *p* = 0.01), and GGT (128.09 ± 157.49 vs. 74.58 ± 82.16 U/L; *p* = 0.003). In addition, the NAS was significantly higher in Group 2 (5.27 ± 1.70 vs. 4.07 ± 1.50; *p* < 0.001), as were the hepatocellular inflammation (1.75 ± 0.90 vs. 1.12 ± 0.72; *p* < 0.001), ballooning (1.57 ± 0.64 vs. 1.09 ± 0.71; *p* < 0.001), and fibrosis (2.83 ± 0.71 vs. 0.60 ± 0.68; *p* < 0.001) scores (Table 1).

**Figure 1:**
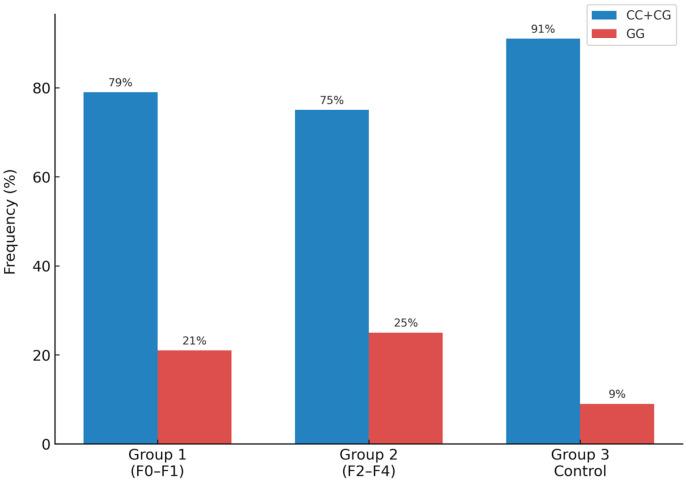
Frequency of the rs738409 polymorphism of the PNPLA3 gene in Groups 1, 2, and 3. GG genotype was significantly higher in Groups 1 and 2 compared to Group 3 (*p* = 0.02).

Table 2 shows the distribution (n) and frequency (%) of the genotypes of the rs738409 polymorphism in the PNPLA3 gene, comparing Groups 1 (F0–F1), 2 (F2–F4) and 3 (control). The CC and CG genotypes were predominant in patients in Groups 1 and 2, with frequencies of 79% and 75%, respectively, and there was no statistically significant difference between Groups 1 and 2 for frequency (*p* > 0.05). However, the frequency of the GG genotype was significantly higher in Groups 1 and 2 (21% and 25%, respectively) compared to Group 3 (9%; *p* = 0.02) (Figure 1).

**Figure 2:**
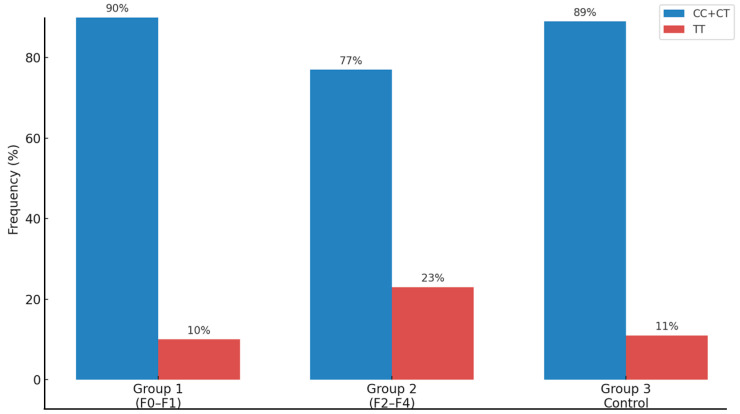
Frequency of the rs641738 polymorphism of the MBOAT7 gene in Groups 1, 2, and 3. TT risk genotype was statistically higher in Group 2 than in Groups 1 and 3 altogether.

The distribution (n) and frequency (%) of the genotypes of the rs641738 polymorphism in the MBOAT7 gene are also shown in Table 2. The CC/CT genotypes were observed in 90% of patients in Group 1 and 77% in Group 2. In the control group (Group 3), the frequency was 89%. The prevalence of the TT risk genotype was statistically higher in Group 2 (23%) than in Groups 1 (10%) and 3 (11%) altogether (*p* = 0.01) (Figure 2).

**Figure 3:**
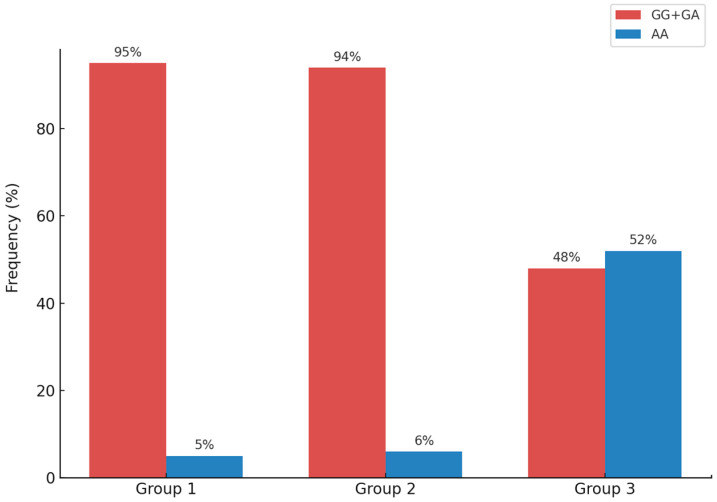
Frequency of the rs2642438 polymorphism of the MTARC1 gene in Groups 1, 2, and 3. AA protective genotype was found in only 5% and 6%, respectively, of patients in Groups 1 and 2, but was more frequent in Group 3 (52%).

The same table also shows the distribution (n) and frequency (%) of the genotypes of the rs2642438 polymorphism in the MTARC1 gene. The GG/GA genotypes were prevalent in patients in Groups 1 and 2 (95% and 94%, respectively), while in the control group (Group 3), this frequency was significantly lower (48%; *p* = 2.76 × 10^−20^). The AA protective genotype was found in only 5% and 6%, respectively, of patients in Groups 1 and 2, but was more frequent in Group 3 (52%) (Figure 3).

The distribution (n) and frequency (%) of the genotypes of the rs72613567 polymorphism in the HSD17B13 gene are shown. The TT genotype was prevalent in all groups, with frequencies of 74% in Group 1, 76% in Group 2, and 65% in Group 3 (*p* = 0.14). The protective TTA/TATA genotypes were observed in 26% of patients in Group 1, 24% in Group 2, and 35% in Group 3, but there was no statistically significant difference between the groups. In fact, the risk variants of the polymorphisms mentioned above were more prevalent in the at-risk population, but we did not obtain statistical significance.

Table 2 shows the distribution (n) and frequency (%) of the rs58542926 polymorphism genotypes in the TM6SF2 gene. In Groups 1 and 2, the CC/CT genotypes were the most common, with frequencies of 98% and 99%, respectively, and there was no statistically significant difference between Groups 1 and 2 (*p* > 0.05). In Group 3 (control), all the individuals had the CC/CT genotype (100%). The TT risk genotype was infrequent, present in only 2% and 1% of patients in Groups 1 and 2, and absent in Group 3, with no statistically significant difference between the groups (*p* = 0.45) (Table 2).

The CC/CT genotypes of the rs1260326 polymorphism in the GCKR gene were tested in 84% of patients in Group 1 and 76% in Group 2, and there was no statistically significant difference between Groups 1 and 2 (*p* = 0.25). In Group 3, the frequency was similar, at 83%, showing no significant difference between the groups (*p* = 0.25). The TT risk genotype was more frequent in Group 2 (24%) compared to Groups 1 (16%) and 3 (17%), but this difference was not significant either (Table 2).

Finally, the CC/CT genotypes of the rs780094 polymorphism in the GCKR gene were predominant in all groups: 85% in Group 1, 78% in Group 2, and 82% in Group 3. The TT risk genotype was more frequent in Group 2 (22%), followed by Groups 1 (15%) and 3 (18%). However, there was no statistically significant difference between the groups (*p* = 0.40) (Table 2).

### Impact of the rs641738 Variant of the MBOAT7 Gene on Significant Fibrosis in Patients with MASLD

The logistic regression model to predict significant fibrosis was built based on the TT genotype of MBOAT7, associated with clinical and biochemical variables, including age, ALT, AST, GGT, TG, HDL, LDL, and total cholesterol.

**Figure 4:**
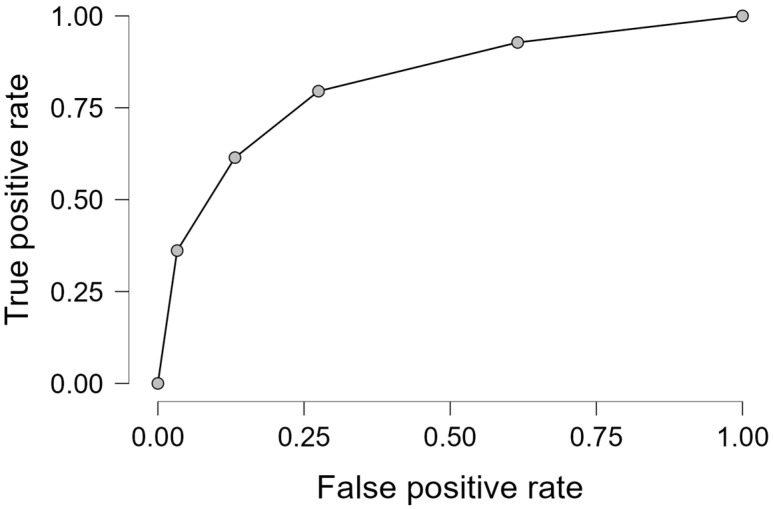
ROC (Receiver Operating Characteristic) evaluating the model’s predictive capacity: the rs641738 (MBOAT7-TT) polymorphism showed AUC = 0.83; sensitivity = 75%; specificity = 81%; accuracy = 78%; and Χ^2^ = 65.33.

A ROC (Receiver Operating Characteristic) curve was used to evaluate the model’s predictive capacity. The rs641738 (MBOAT7-TT) polymorphism showed a high AUC (Area Under the Curve) of 0.83, indicating that it is a predictor of liver fibrosis (AUC = 0.83; sensitivity = 75%; specificity = 81%; accuracy = 78%; Χ^2^ = 65.33) (Figure 4).

## 3. Discussion

The global prevalence of MASLD is estimated at 30%, and more than 20% of cases can progress to more serious conditions. Previous studies, such as those by Bianco et al. (2021), have already demonstrated the relevance of non-invasive methods for stratifying the risk of patients with HCC resulting from MASLD [13]. Our study complements this literature by focusing on fibrosis stages and highlighting genetic factors as predictors of disease progression. In addition, this is the first study to investigates the relationship between the rs738409 (PNPLA3), rs58542926 (TM6SF2), rs1260326 and rs780094 (GCKR), rs641738 (MBOAT7), rs72613567 (HSD17B13), and rs2642438 (MTARC1) polymorphisms in the Brazilian population with MASLD and hepatic fibrosis.

The PNPLA3 gene, often associated with elevated liver enzymes and the progression of MASLD, confirmed its pro-fibrotic effect in our study [12]. The GG genotype of the rs738409 polymorphism was more prevalent in Groups 1 (F0–F1) and 2 (F2–F4), with 23% and 22%, respectively, compared to the control group (9%, *p* = 0.02). These findings reinforce that PNPLA3 is an important genetic marker in patients with MASLD in the Latin American population.

The genotype distribution of rs738409 (PNPLA3) varies globally, reflecting ethnic differences and possible environmental influences. Population data indicate the following genotype frequencies by continent:

- Europeans: CC (46%), CG (32%), and GG (22%);
- Asians: CC (18%), CG (42%), and GG (40%);
- Africans: CC (96%), CG (3.9%), and GG (0.1%);
- Latin Americans (Brazil): CC (46.8%), CG (40.5%), and GG (12.6%);
- Chinese: CC (39.2%), CG (47.6%), and GG (13.2%).

These data reinforce that the GG genotype has an intermediate value in the Brazilian population, which is indeed a mixture of Europeans, Africans, and South American Indians, which may influence susceptibility to MASLD and its complications in our population [14,15,16,17,18].

Some studies have shown that the homozygous recessive GG is more present in individuals with high BMIs and advanced ages, making them more susceptible to the progression of MASLD, especially in women [17]. In our study, the population was predominantly female (Group 1: 68.14%; Group 2: 69.70%), with a high average BMI of 31.5 and an age over 50 (Group 1: 52.8 years; Group 2: 55.7 years).

In addition, recent studies have highlighted the impact of rs738409 on so-called lean MASLD (BMI < 25), suggesting that the polymorphism may act as a risk factor independent of overweight [19]. Evidence from the UK Biobank indicates that the interaction between the PNPLA3 genotype, alcohol intake, and obesity can increase the risk of HCC by up to 30 times [19,20].

The MBOAT7 rs641738 T polymorphism has a variable genotype distribution among different populations. The highest frequencies of the TT risk genotype are observed in South Asian (28%) and European (20%) populations, while the lowest occur in East Asian populations, such as the Japanese (4%) and Taiwanese (4%) populations [21]. Otherwise, in an Italian population (South Europe), the TT frequency was 19%, and in a United Kingdom population (North Europe), it was 20% [22], showing that there is no difference between southern and northern regions. The global genotypic distribution of the rs641738 (C>T) polymorphism indicates the following:

- Europeans: CC (35%), CT (45%), and TT (20%);
- Latin Americans (Brazil): CC (44%), CT (46%), and TT (10%);
- Africans: CC (46%), CT (44%), and TT (10%);
- East Asia: CC (32%), CT (49%), and TT (19%);
- South Asia: CC (22%), CT (50%), and TT (28%);
- South Europe: CC (32%), CT (50%), and TT (19%);
- North Europe: CC (33%), CT (47%), and TT (20%).

In our study, we confirmed that carriers of the TT genotype of the rs641738 polymorphism in the MBOAT7 gene have a 5.02 times greater risk of developing liver fibrosis above grade 2, corroborating the findings of Bianco et al. (2021). This polymorphism showed a highly significant risk effect on fibrogenesis, with an OR of 9.2 in the NAFLD/MASLD cohort (*p* = 2.7 × 10^−14^) [13].

The MBOAT7 gene encodes the enzyme lysophosphatidylinositol acyltransferase, which is essential in the phospholipid remodeling cycle, incorporating polyunsaturated fatty acids into cell membranes. The rs641738 C>T variant, which replaces the amino acid glutamate with valine, reduces the activity of the gene, compromising phospholipid metabolism and contributing to the abnormal retention of triglycerides in the liver [23,24].

The higher prevalence of the risk genotype (TT) in patients with significant fibrosis reinforces its association with the progression of liver disease, in line with data in the literature.

In addition to individual associations, we explored the potential combined effect of PNPLA3 and MBOAT7 variants on liver fibrosis using a logistic regression model that included both polymorphisms. Although the individual associations remained significant, no synergistic or additive interaction was observed (*p* > 0.05). This lack of significance may be due to limited sample size or effect heterogeneity in our admixed cohort. While previous studies have reported additive risk when both alleles are present, particularly in European populations, our data suggest that the influence of these variants on fibrosis may be independent in this Brazilian population. Further studies with larger sample sizes and polygenic risk modeling may help to clarify gene–gene interactions in MASLD.

The recent literature has provided compelling evidence on the potential mechanisms linking these genetic variants to hepatic fibrogenesis. The MBOAT7 rs641738 T allele has been shown to downregulate the expression of lysophosphatidylinositol acyltransferase 1 (also known as LPIAT1), impairing phospholipid remodeling and resulting in the accumulation of pro-inflammatory lipotoxic species [22,24].

In contrast, the protective MTARC1 rs2642438 AA genotype has been associated with improved mitochondrial function, reduced production of reactive oxygen species (ROS), and attenuation of lipid peroxidation in hepatocytes. MTARC1 encodes an enzyme involved in mitochondrial redox reactions; its protective allele appears to reduce oxidative stress and improve metabolic flexibility, contributing to a less fibrogenic hepatic environment [12,25].

Furthermore, the PNPLA3 rs738409 GG variant results in a dysfunctional protein with impaired triglyceride hydrolysis, leading to lipid droplet accumulation. This lipotoxic environment may indirectly stimulate hepatic stellate cells and fibrogenic signaling cascades, especially in the context of metabolic syndrome and insulin resistance [14].

On the other hand, the rs2642438 polymorphism in the MTARC1 gene showed a significant protective effect against liver fibrosis [5]. In our analysis, the AA genotype, predominant in the control group (52%), was associated with a lower prevalence of fibrosis, while the GG and GA genotypes were more frequent among patients with significant fibrosis. This finding suggests that the protective genotype attenuates fibrogenesis processes, possibly due to its influence on hepatic lipid composition. The global genotype distribution of rs2642438 (G>A) in MTARC1 indicates the following:

- Europeans: AA (53%), AG (39%), and GG (8%);
- African-Americans: AA (90%), AG (9%), and GG (1%);
- Asians: AA (95%), AG (5%), and GG (0%).

These data suggest that the G allele (associated with the risk of fibrosis) is more frequent in European population, while it has a low prevalence in African Americans (1%) and Asians (0%), suggesting possible differences in genetic susceptibility to liver fibrosis between populations [26]. Data on this gene are still scarce in admixed or Latin American populations.

Recent studies, such as that by Jones et al. (2024), have reinforced the protective role of MTARC1, showing that carriers of the rs2642438 variant in murine models with MASH showed weight loss, reduced hepatic steatosis, lower oxidative stress, and reduced fibrogenesis markers [25]. Similar results were reported by Guo Yuanjun et al. (2024), who demonstrated a decrease in serum liver enzymes, LDL cholesterol, and triglycerides in rat models of MASLD, as well as an improvement in the lipid profile and attenuation of pathological changes in the liver [27].

Although no significant associations were observed between liver fibrosis and the TM6SF2, GCKR, or HSD17B13 polymorphisms in our cohort, these variants have been reported to influence MASLD susceptibility in other populations. Several factors may explain this discrepancy. First, our sample size, although adequate for the primary associations found, may have limited power to detect modest effect sizes for other variants. Second, the Brazilian population is highly admixed, with varying proportions of European, African, and Indigenous ancestry, which may influence allele frequencies, linkage disequilibrium patterns, and gene–environment interactions. Lastly, lifestyle factors, dietary patterns, and coexisting metabolic traits (e.g., insulin resistance, obesity, alcohol intake) could modulate the phenotypic impact of these variants.

Future studies should include a larger number of participants and longitudinal analyses to better understand the causality between polymorphisms and MASLD. In addition, multicenter studies involving different populations in Brazil could increase the robustness of the conclusions. The inclusion of environmental variables could also contribute to a more comprehensive analysis of the disease.

Despite the limitations, this study has important positive points. The work contributes significantly to understanding the role of genetic polymorphisms in liver fibrosis associated with MASLD, highlighting the relevance of the MTARC1 gene as a protective factor. In addition, the findings reinforce the importance of genotyping as a potential tool for risk stratification and the personalization of clinical management.

One important limitation of this study is the use of blood donors as the control group. Although they were screened to exclude known liver disease and viral hepatitis, they were not evaluated through imaging or liver function scoring to definitively rule out subclinical MASLD. Additionally, due to ethical constraints and compliance with the Brazilian General Data Protection Law (LGPD), only limited demographic data (age, sex, and BMI) were available for this group. Blood donors also tend to be younger, have lower BMIs, and are generally healthier than the general population. These differences were reflected in our data, where the control group showed a significantly lower age and BMI profile compared to MASLD patients. This selection bias may affect the comparability between groups and could potentially lead to an overestimation of genetic associations.

However, since the primary outcome of interest was the presence of specific SNPs—rather than biochemical or histological markers—and given that genetic variants are inherited and stable throughout life, we believe the observed associations remain valid. Nevertheless, differences in age and sex distribution may introduce residual confounding. Therefore, caution is warranted when extrapolating our findings to broader or more diverse populations. Future studies using age- and sex-matched, population-based controls with complete clinical and metabolic profiles are recommended to enhance external validity.

The current MASLD nomenclature does allow for modest alcohol use, but distinguishing metabolic from alcohol-induced liver injury can be complex, particularly in admixed populations. In our cohort, all patients classified as MASLD had alcohol consumption below established exclusion thresholds, based on clinical interviews and medical records. While our genetic assays were not specifically designed to differentiate between metabolic and alcohol-related liver disease, emerging evidence suggests that certain variants—particularly PNPLA3 rs738409 and MBOAT7 rs641738—are enriched in both MASLD and alcoholic liver disease.

## 4. Materials and Methods

This cross-sectional, retrospective, unicentric study analyzed DNA blood samples from patients with MASLD and individuals without a diagnosis of MASLD by liver biopsy. This study was carried out at the Hospital das Clínicas e Laboratório de Gastroenterologia e Clínica e Experimental (LIM07) da Faculdade de Medicina da Universidade de São Paulo.

### 4.1. Study Population

In total, 212 patients with a histological diagnosis of MASLD and 90 individuals without a diagnosis of liver disease, used as a control group, were included. The control group samples were obtained from blood donors from the Fundação Pró-Sangue Hemocentro de São Paulo.

### 4.2. Inclusion Criteria

The inclusion criteria were as follows: patients with a diagnosis of MASLD confirmed by liver biopsy, individuals over the age of 18 with informed consent for the use of biological material, and individuals in the control group with no history of liver disease, hepatotropic viral infections, or excessive alcohol consumption. Laboratory data were collected up to 6 months before or after the baseline date, which corresponds to the date of the first liver biopsy, performed as a diagnostic biopsy.

The exclusion criteria were samples with incomplete data or without adequate histopathological information and individuals with liver diseases of other etiologies.

### 4.3. Analyses

DNA was extracted from peripheral blood samples using the QIAamp DNA Blood Mini kit (QIAGEN, São Paulo, Brazil), following the manufacturer’s instructions. DNA quantification and purity were determined by spectrophotometry (NanoDrop 1000, Thermo Fisher Scientific, Wilmington, DE, USA). The samples were then diluted according to the manufacturer’s instructions.

The polymorphisms studied were genotyped by real-time PCR (qPCR) using TaqMan probes. The analyses were carried out on 7500 Fast Real-Time PCR equipment (Applied Biosystems, Foster City, CA, USA). The following polymorphisms were evaluated: PNPLA3 C>G (rs738409), TM6SF2 C>T (rs58542926), GCKR C>T (rs1260326 and rs780094), MBOAT7 C>T (rs641738), HSD17B13:T (rs72613567), and MTARC1 A>G (rs2642438). PCR was performed according to the following program: 95 °C for 10 min, followed by 40 cycles of denaturation at 95 °C for 15 s, annealing at 60 °C for 1 min, and extension at 60 °C for 1 min.

Statistical analysis was carried out using the JASP program version 0.18 (JASP Team, Amsterdam University, Netherlands). For continuous quantitative variables, the difference between means was checked using the Student’s *t*-test; the association between nominal qualitative variables was tested using Chi-square. The significance level adopted was 5%. A logistic regression model was developed to assess the predictors of fibrosis. Variables with *p* < 0.2 in the univariate analysis were included in the model. Regression was carried out using the “enter” method. Nagelkerke’s R2 was used to determine the proportion of variation explained by the model. The results of the multivariate analysis are presented as odds ratios (ORS) with their respective 95% confidence intervals (95% CIs). The sample size was not calculated, and patients and controls were included based on convenience.

Patients with MASLD were classified into two groups based on the degree of liver fibrosis, according to histological assessment: Group 1 (F0/F1)—patients without significant fibrosis; and Group 2 (F2/F3/F4)—patients with significant fibrosis. The control group consisted of blood donors without a diagnosis of liver disease.

## 5. Conclusions

The findings of this study show that the PNPLA3 (rs738409), MBOAT7 (rs641738), and MTARC1 (rs2642438) polymorphisms are associated with MASLD in the Brazilian population.

The rs738409 polymorphism in the PNPLA3 gene was significantly more frequent in patients with MASLD compared to the general population, reinforcing its role as a genetic risk biomarker. Similarly, the rs641738 polymorphism in the MBOAT7 gene was associated with an increased risk of fibrosis in MASLD, potentially due to its role in lipid metabolism and liver fibrogenesis. However, given the cross-sectional design of this study, these findings should be interpreted as associations rather than evidence of causality. In contrast, the rs2642438 polymorphism in the MTARC1 gene showed a protective association, suggesting a possible role in modulating liver damage.

The results of this study reinforce the importance of genetic polymorphisms in the progression of MASLD, highlighting significant associations between patients with different degrees of fibrosis and individuals in the control group, with no previous diagnosis of the disease. This is the first study to investigate the relationship between the PNPLA3 rs738409, TM6SF2 rs58542926, GCKR rs1260326 and rs780094, MBOAT7 rs641738, HSD17B13 rs72613567, and MTARC1 rs2642438 polymorphisms in the Brazilian population with MASLD and liver fibrosis. These findings underscore the potential relevance of genotyping as a complementary tool in MASLD risk stratification. Nevertheless, prospective and functional studies are required to confirm the causal role of these variants. Further studies are needed to validate these associations in admixed populations and to explore potential interactions with metabolic and environmental factors.

## Figures and Tables

**Table 1:** Demographic, clinical, and biochemical characteristics of patients with metabolic dysfunction-associated steatotic liver disease (MASLD).

Characteristic	Group 1 n = 110 (F0–F1)	Group 2 n = 102 (F2–F4)	*p*
Age, years	52.8 ± 10.91	55.7 ± 11.46	<0.05
Sex (F/M) %	68.14/31.86	69.70/30.30	0.19
BMI, kg/m^2^	32.29 ± 6.49	30.8 ± 5.98	<0.05
DLP (Yes/No)	56/42	38/52	0.06
T2D (Yes/No)	59/39	62/28	0.28
Hypertension (Yes/No)	70/28	64/26	1.00
Glucose, mg/dL	114.7 ± 37.52	129.30 ± 54.85	<0.05
Insulin, µU/mL	21.36 ± 16.30	20.72 ± 12.33	0.83
AST (U/L)	31.95 ± 26.90	49.81 ± 40.24	<0.05
ALT (U/L)	44.34 ± 35.72	58.16 ± 42.26	<0.05
GGT (U/L)	74.58 ± 82.16	128.09 ± 157.49	<0.05
TC (mg/dL)	197.77 ± 40.85	180.27 ± 44.77	<0.05
HDL-C (mg/dL)	48.16 ± 15.32	44.34 ± 11.74	0.06
LDL-C (mg/dL)	115.26 ± 37.37	106.63 ± 37.60	0.12
TG (mg/dL)	184.13 ± 198.36	187.58 ± 180.5	0.90
AFP (ng/mL)	2.40 ± 1.13	3.81 ± 2.58	<0.05
MASH	74	84	-
NAS (0–8)	4.07 ± 1.50	5.27 ± 1.70	<0.05
Steatosis (0–3)	0: 7/1: 29/2: 45/3: 29	0: 5/1: 28/2: 37/3: 32	-
Inflammation (0–3)	0: 20/1: 58/2: 30/3: 2	0: 9/1: 32/2: 39/3: 22	-
Balloonization (0–2)	0: 23/1: 55/2: 32	0: 9/1: 27/2: 66	-
Fibrosis (0–4)	0: 52/1: 58	2: 35/3: 48/4: 19	-

Values are presented as means ± standard deviations. BMI, body mass; DLP, dyslipidemia; AST, aspartate transaminase; ALT, alanine aminotransferase; GGT, gamma-glutamyl transpeptidase; TC, total cholesterol; HDL-C, high-density lipoprotein cholesterol; LDL-C, low-density lipoprotein cholesterol; TG, triacylglyceride; AFP, alphafetroprotein; MASH, metabolic dysfunction-associated steatohepatitis; NAS, NAFLD activity score. Reference values: glucose (70 to 99 mg/dL); insulin (2.6–24.9 µU/mL); AST: male (<37 U/L), female: (<31 U/L); ALT: male: (<41 U/L), female: (<31 U/L); GGT: male: (8–61 U/L), female: (5–36 U/L); total cholesterol: desirable (<to 200 mg/dL), borderline: (200–239 mg/dL), high: (≥240 mg/dL); HDL: no risk: (>65 mg/dL), moderate risk: (45 to 65 mg/dL), high risk: (<45 mg/dL); LDL: (no risk: <130 mg/dL), moderate risk: (130 to 159 mg/dL), high risk: (>159 mg/dL); TG: desirable: (<150 mg/dL), borderline: (150 to 200 mg/dL), high: (200 to 499 mg/dL), very high: (≥500 mg/dL). Note: clinical, biochemical, and histological data were not available for Group 3 (control group), as these individuals were healthy blood donors, and further data access was restricted in accordance with the Brazilian General Data Protection Law (LGPD).

**Table 2:** Distribution (n) and frequency (%) of the genotypes of the rs738409 PNPLA3, rs641738 MBOAT7, and rs2642438 MTARC1 polymorphisms in patients with metabolic dysfunction-associated steatotic liver disease (MASLD) and the control group.

Gene Polymorphism	Genotype	Group 1 (F0–F1)	Group 2 (F2–F4)	Group 3 (Control Group)	*p*
PNPLA3 rs738409	CC/CG (%)	87 (79%)	77 (75%)	82 (91%)	<0.05
	GG (%)	23 (21%)	25 (25%)	8 (9%)	
MBOAT7 rs641738	CC/CT (%)	99 (90%)	79 (77%)	80 (89%)	<0.05
	TT (%)	11 (10%)	23 (23%)	10 (11%)	
MTARC1 rs2642438	GG/GA (%)	105 (95%)	96 (94%)	43 (48%)	<0.05
	AA (%)	5 (5%)	6 (6%)	47 (52%)	
TM6SF2 rs58542926	CC/CT (%)	108 (98%)	101 (99%)	90 (100%)	>0.05
	TT (%)	2 (2%)	1 (1%)	0 (0%)	
GCKR rs1260326	CC/CT (%)	92 (83%)	78 (76%)	75 (83%)	>0.05
	TT (%)	18 (17%)	24 (24%)	15 (17%)	
GCKR rs780094	CC/CT (%)	963 (84%)	80 (78%)	74 (82%)	>0.05
	TT (%)	17 (16%)	22 (22%)	16 (18%)	
HSD17B13 rs72613567	TT (%)	82 (74%)	78 (76%)	58 (65%)	>0.05
	TTA/TATA (%)	28 (26%)	24 (24%)	32 (35%)	

References: PNPLA3—CC (homozygous dominant), CG (heterozygous), GG (homozygous recessive—risk genotype); TM6SF2, GCKR, MBOAT7—CC (homozygous dominant), CT (heterozygous), TT (homozygous recessive—risk genotype); MTARC1—GG (homozygous dominant), GA (heterozygous), AA (homozygous recessive—protective genotype); HSD17B13—TT (homozygous dominant), TTA (heterozygous—protective genotype), TATA (homozygous recessive—rare and protective genotype).

## Data Availability

The sources of all underlying data and modeling assumptions are available upon request from the corresponding author.

## Abbreviations

The following abbreviations are used in this manuscript:

AFP	Alpha-Fetoprotein
ALT	Alanine Aminotransferase
AST	Aspartate Transaminase
BMI	Body Mass
DLP	Dyslipidemia
GCKR	Glucokinase Regulator
GGT	Gamma-Glutamyl Transpeptidase
HCC	Hepatocellular Carcinoma
HCFMUSP	Hospital Das Clínicas Da Faculdade De Medicina Da Universidade De São Paulo
HDL-C	High-Density Lipoprotein Cholesterol
HSD17B13	Hydroxysteroid 17-Beta Dehydrogenase 13
LDL-C	Low-Density Lipoprotein Cholesterol
MASLD	Metabolic Dysfunction-Associated Steatotic Liver Disease
MASH	Metabolic Dysfunction-Associated Steatohepatitis
MBOAT7	Membrane Bound O-Acyltransferase Domain Containing 7
MTARC1	Mitochondrial Amidoxime Reducing Component 1
NAS	NAFLD Activity Score
PNPLA3	Patatin Like Phospholipase Domain Containing 3
SNPs	Single Nucleotide Polymorphisms
TC	Total Cholesterol
T2DM	Type 2 Diabetes
TG	Triacyl Glyceride
TM6SF2	Transmembrane 6 Superfamily Member 2
USP	Universidade de São Paulo
